# Can States Simultaneously Improve Health Outcomes and Reduce Health Outcome Disparities?

**DOI:** 10.5888/pcd13.160126

**Published:** 2016-08-25

**Authors:** David Kindig, Nicholas Lardinois, Debanjana Chatterjee

**Affiliations:** Author Affiliations: Nicholas Lardinois, University of Wisconsin-Madison, Population Health Institute, Madison, Wisconsin; Debanjana Chatterjee, Division of General Pediatrics and Adolescent Health, University of Minnesota School of Medicine, Minneapolis, Minnesota.

## Abstract

**Introduction:**

Reducing racial health disparities is often stated as a population health goal, but specific targets for such improvement are seldom set. It is often assumed that improving overall health outcomes will be linked to disparity reduction, but this is not necessarily the case.

**Methods:**

We compared the annual change from 1999 through 2013 in combined-race (black and white) mortality with the annual change in absolute and relative racial mortality disparities for US states.

**Results:**

Median annual improvement in combined-race mortality was 1.08% per year. Annual overall mortality rate reductions ranged from 0.24% per year in Oklahoma to 1.83% per year in Maryland. For disparities, the median for the black–white absolute gap was 3.60% per year, and the median for the relative black-to-white ratio was 1.19% per year. There was no significant correlation between the combined-race measure and either the absolute (0.03) or relative disparity measure reductions (−0.17).

**Conclusion:**

For mortality in US states over a recent period, improvement in the population mean and disparity reduction do not usually occur together. The disparity reduction rates observed may provide realistic guidance for public and private policy makers in setting goals for reducing population health disparity and creating investment priorities. As a starting point for discussion, the observed national median annual percentage improvement of 1.1 per year combined, 3.6% per year absolute gap reduction, and 1.2% per year relative gap reduction would be modest and reasonable goals.

## Introduction

National and state health outcome goals are often framed in terms of improving the population mean and reducing or eliminating disparities within the population. For example, in *Healthy People 2020*, the 2 overarching goals are 1) attain high-quality, longer lives free of preventable disease, disability, injury, and premature death, and 2) achieve health equity, eliminate disparities, and improve the health of all groups (1). However as Keppel et al pointed out with regard to *Healthy People 2010,* the first goal does not necessarily achieve health equity, eliminate disparities, and improve the health of all groups (2). Different strategies are often needed for these 2 goals, and innovations often have higher effect, at least initially, on well-educated or advantaged populations, which can at least temporarily increase disparities (2–4).

Trends in state health outcomes show large variations over time (5–8). Satcher et al showed that the black–white gap in mortality rates changed little between 1960 and 2000 (9). Another recent study found that large racial disparities in some states are explained by higher-than-average life expectancy among whites or lower-than-average life expectancy among blacks (10). Webb et al constructed a health disparity index by race, which compared state performance but did not contrast disparity reduction with mean improvement (11).

To our knowledge no jurisdiction in the United States has emphasized the potential trade-off between these 2 outcomes or given policy attention to how such trade-offs should be addressed. The objective of this study was to describe what US states recently experienced in overall mean improvement in mortality compared with the improvement in the black–white mortality gap. We tested the hypothesis that states that experience the greatest improvements in combined-race mortality also experience the greatest improvements in reducing racial disparities. We hope that such evidence will guide policy and investment planning so that the United States and the 50 individual states can set reasonable annual improvement targets to achieve in the coming decades.

## Methods

Data on age-adjusted mortality come from the publicly available CDC Wonder’s Compressed Mortality Database (12). We extracted mortality data on people younger than 75 years for all 50 US states and Washington, DC, for all sexes, for blacks, and for white non-Hispanics for all years from 1999 through 2013. Mortality was calculated per 100,000 people and age-adjusted by using the 2000 US standard population.

We calculated the annual percentage change for the combined mortality of both races as well as by 2 mortality disparity measures for each state: racial gap as an absolute disparity measure and the black-to-white ratio as a relative disparity measure. Absolute disparity refers to the simple difference in a health outcome, whereas relative disparity refers to the health outcome of one group as a ratio of the other’s health outcome. To generate annual percentage changes, we used Stata/SE 14.0 (StataCorp LP) to predict values from a linear regression of each measure on years for each state, providing a smooth linear trend (13).

The absolute mortality gap by race was calculated by subtracting the white age-adjusted mortality from the black age-adjusted mortality for each year and each state. States that reported 50 or fewer deaths in 2000 in either racial category were excluded from our analysis because too few events can cause large variation in year-to-year mortality; for blacks, the states excluded were Idaho, Maine, Montana, New Hampshire, North Dakota, South Dakota, Vermont, and Wyoming. Also excluded from our analysis were 4 states with a statistically insignificant relationship between the age-adjusted mortality racial gap and year according to a linear regression model of age-adjusted mortality racial gap and year: the excluded states were Alaska, Hawaii, West Virginia, and Wisconsin. If a basic linear regression reveals statistically insignificant results, we cannot conclude that these states’ disparity reductions were statistically different from zero.

Recently published research results note that decisions about measuring disparities using absolute or relative methods, such as group rankings and direction and magnitude of changes over time, has an effect on results (14–16). Therefore, we follow the recommendation to examine and report both absolute and relative disparity results. The relative disparity measure we examined was a black-to-white ratio of age-adjusted mortality for each state. Five additional states were found not to have significant annual percentage changes in relative mortality disparity and therefore were excluded from the relative-disparity portion of our analysis: Colorado, Iowa, Nebraska, New Mexico, and Utah.

## Results

**Figure 1:**
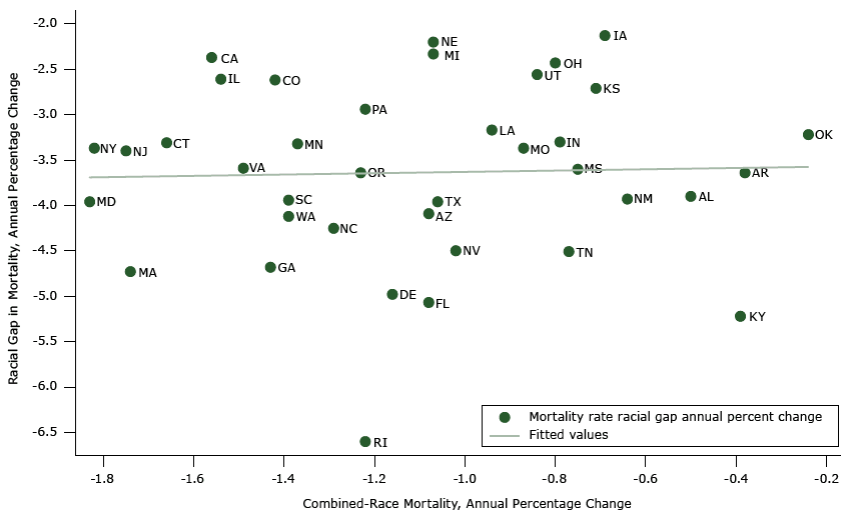
Racial gap between blacks and whites versus annual percentage change in combined-race mortality: variation in 38 states’ annual percentage improvement in combined-race mortality and in absolute racial gaps, 1999–2013. **Table:** 

The Table describes 1) the relationship across the states examined between annual state percentage improvement in mortality and 2) the change in the absolute racial gap and relative racial disparity from 1999 through 2013. The mean combined-race mortality rate of annual improvement was 1.11%, (standard deviation [SD] 0.42%). The range was from 1.83% in Maryland to 0.24% in Oklahoma. For annual percentage change in the racial gap, the mean was a reduction of 3.64% per year (SD, 0.97%). The range was from 6.6% in Rhode Island to 2.13% in Iowa. For annual percentage change in the black-to-white ratio, the mean was an annual reduction of 1.2% (SD, 0.4%). State mortality varied from 2.47% in Rhode Island to 0.43% in California. Figure 1 displays findings for the absolute disparity measure for all states examined.

**Table:** State Annual Percentage Variation in Improvement in Combined-Race (Black and White) Mortality^a^, Absolute Racial Gap, and Relative Racial Gap, 1999–2013

Category	Mean	Median	High	Low	Standard Deviation
Combined-race mortality (38 states)	−1.11	−1.08	−1.83	−0.24	0.42
Absolute racial gap (38 states)	−3.64	−3.60	−6.6	−2.13	0.97
Combined-race mortality (33 states)^b^	−1.14	−1.16	−1.83	−0.24	0.43
Relative racial disparity (33 states)^b^	−1.2	−1.19	−2.47	−0.43	0.4

a Mortality was calculated per 100,000 people and age-adjusted by using the 2000 standardized US population.

b Only 33 states were relevant for the method used to measure relative disparity.

State	Combined-Race Mortality, Annual Percentage Change	Racial Gap in Mortality, Annual Percentage Change
Alabama (AL)	−0.5	−3.9
Alaska (AK)	−0.38	−3.64
Arizona (AZ)	−1.08	−4.09
California (CA)	−1.56	−2.37
Colorado (CO)	−1.42	−2.62
Connecticut (CT)	−1.66	−3.31
Delaware (DE)	−1.16	−4.98
Florida (FL)	−1.08	−5.07
Georgia (GA)	−1.43	−4.68
Iowa (IA)	−0.69	−2.13
Illinois (IL)	−1.54	−2.61
Indiana (IN)	−0.79	−3.3
Kansas (KS)	−0.71	−2.71
Kentucky (KY)	−0.39	−5.22
Louisiana (LA)	−0.94	−3.17
Massachusetts (MA)	−1.74	−4.73
Maryland (MD)	−1.83	−3.96
Michigan (MI)	−1.07	−2.33
Minnesota (MN)	−1.37	−3.32
Missouri (MO)	−0.87	−3.37
Mississippi (MS)	−0.75	−3.6
North Carolina (NC)	−1.29	−4.25
Nebraska (NE)	−1.07	−2.2
New Jersey (NJ)	−1.75	−3.4
New Mexico (NM)	−0.64	−3.93
Nevada (NV)	−1.02	−4.5
New York (NY)	−1.82	−3.37
Ohio (OH)	−0.8	−2.43
Oklahoma (OK)	−0.24	−3.22
Oregon (OR)	−1.23	−3.64
Pennsylvania (PA)	−1.22	−2.94
Rhode Island (RI)	−1.22	−6.6
South Carolina (SC)	−1.39	−3.94
Tennessee (TN)	−0.77	−4.51
Texas (TX)	−1.06	−3.96
Utah (UT)	−0.84	−2.56
Virginia (VA)	−1.49	−3.59
Washington (WA)	−1.39	−4.12

The extent of improvement in combined mortality was not correlated with the reduction in the racial mortality gap (correlation coefficient = 0.03). Some states (eg, Massachusetts, Georgia) improved substantially on both combined-race mortality and absolute racial gap outcomes while other states’ (eg, Oklahoma, Iowa) improvement on both was not as great. Similarly, states (eg, California, Illinois) did well on combined-race mortality improvement but not as well on absolute racial gap improvement. Conversely, some states (eg, Kentucky, Tennessee) failed to have great improvements in combined-race mortality but saw a large reduction in the absolute racial gap from 1999–2013. In every state there was improvement in both black and white mortality, but improvement in black mortality was always greater (data not shown).

**Figure 2:**
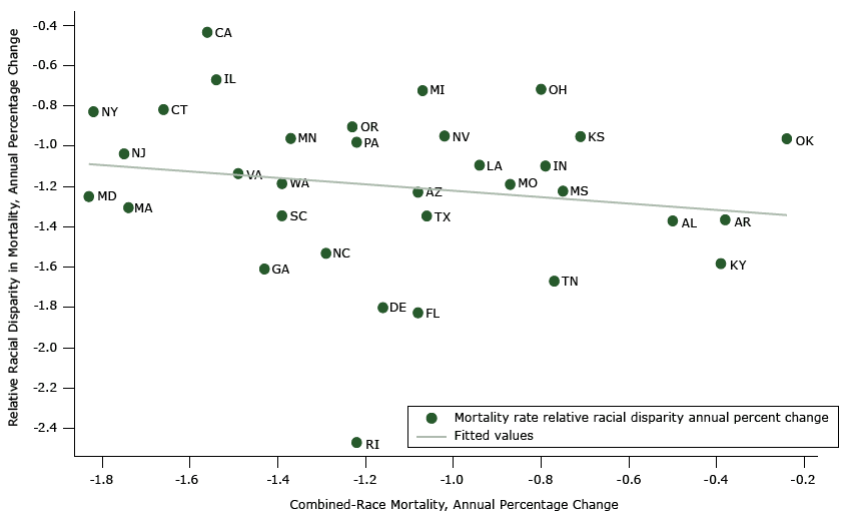
Relative racial disparity versus annual percentage change in combined-race mortality: variation in 33 states’ annual percentage improvement in combined-race (black and white) mortality and relative racial disparities, 1999–2013. **Table:** 

Figure 2 displays a similar relationship between relative disparity and annual percentage changes in combined-race mortality across states. Again, the extent of state improvement in combined mortality is not correlated with the reduction in the relative black-to-white ratio disparity measure (correlation coefficient = −0.17). Massachusetts and Maryland improved substantially on both combined-race mortality and relative racial gap outcomes while other states’ (eg, Oklahoma, Kansas) improvement on both was not as great. California and Illinois improved greatly on the combined-race mortality rate but not as well on the relative black-to-white ratio; the opposite was true for Kentucky and Tennessee.

State	Combined-Race Mortality, Annual Percentage Change	Relative Racial Disparity in Mortality, Annual Percentage Change
Alabama (AL)	−0.5	−1.37
Alaska (AK)	−0.38	−1.37
Arizona (AZ)	−1.08	−1.23
California (CA)	−1.56	−0.43
Connecticut (CT)	−1.66	−0.82
Delaware (DE)	−1.16	−1.80
Florida (FL)	−1.08	−1.83
Georgia (GA)	−1.43	−1.61
Illinois (IL)	−1.54	−0.67
Indiana (IN)	−0.79	−1.10
Kansas (KS)	−0.71	−0.95
Kentucky (KY)	−0.39	−1.58
Louisiana (LA)	−0.94	−1.09
Massachusetts (MA)	−1.74	−1.30
Maryland (MD)	−1.83	−1.25
Michigan (MI)	−1.07	−0.72
Minnesota (MN)	−1.37	−0.96
Missouri (MO)	−0.87	−1.19
Mississippi (MS)	−0.75	−1.22
North Carolina (NC)	−1.29	−1.53
New Jersey (NJ)	−1.75	−1.04
Nevada (NV)	−1.02	−0.95
New York (NY)	−1.82	−0.83
Ohio (OH)	−0.8	−0.72
Oklahoma (OK)	−0.24	−0.96
Oregon (OR)	−1.23	−0.90
Pennsylvania (PA)	−1.22	−0.98
Rhode Island (RI)	−1.22	−2.47
South Carolina (SC)	−1.39	−1.34
Tennessee (TN)	−0.77	−1.67
Texas (TX)	−1.06	−1.35
Virginia (VA)	−1.49	−1.14
Washington (WA)	−1.39	−1.19

## Discussion

The lack of a strong relationship between the combined improvement in mortality and improvement in the disparity gaps answers the question posed in the title and in our hypothesis; most states have not achieved these 2 outcomes simultaneously. Some states, such as Massachusetts, did well on both mean mortality improvement and disparity reduction, while others, such as Oklahoma, have had difficulty with both. However, states often perform well with one dimension but struggle with the other.

We believe our data provide some guidance about what is possible for any state to achieve. At least one state, Maryland, experienced an annual improvement in combined mortality by 1.83% from 1999 through 2013; at least one state, Rhode Island, experienced an annual improvement of 6.6% per year in the black–white mortality absolute gap; at least one state, Rhode Island, had an annual improvement of 2.47% per year in the relative black-to-white mortality ratio. These are not theoretical targets; they are results that were achieved by at least one state during the past decade. Massachusetts performed the best in simultaneous overall improvement (1.74%), absolute disparity reduction (4.73%), and relative disparity reduction (1.3%). More work could also model and project realistic overall and disparity reduction targets that the United States and the individual US states could each achieve by using the highest performing states as guides. As a starting point for discussion, the observed national median annual percentage of 1.1% combined improvement, 3.6% absolute gap reduction, and 1.2% relative gap reduction could be future state continuous improvement benchmarks. Some states might want to use these baselines to develop targets, while others might want to use as baselines what peer states accomplished.

Our results reinforce the importance of reporting disparity results in both absolute and relative terms. Although neither measure showed an overall correlation with combined mortality improvement, there were differences across the states depending on the disparity measure used. Although each disparity measure has slightly different results, the annual percentage change for absolute and relative disparity are strongly linked (correlation coefficient = 0.91). Although in this study both measures showed improvement, there are examples where one measure improves but the other does not (17). Since neither is intrinsically preferable from a policy perspective, policy makers should continue to measure and target reductions in both.

Several limitations should be considered when interpreting our results. This analysis is limited to all-cause mortality; additional research is needed to look into age-specific mortality as well as non-mortality outcomes such as morbidity and health-related quality of life measures (18). In addition, specific-age groups should be examined to see whether there are life stage differences in these findings. We limited our analysis to state changes, so we do not know the extent of variation across counties in improvements or declines in trends. Nor did we examine other disparity domains such as socioeconomic status, which should be investigated since health-related socioeconomic disparities are large and are seen in every state (19). It would also be useful to examine other periods to determine whether the range in improvement we found was achieved in other periods, since what occurred in our study period might be an imperfect guide to future possibilities; the mortality experience of the previous cohorts that produced these results may be different in either direction for more recent cohorts.

Of course, these results do not indicate how any state achieved the results that we show here. We use the term “experienced” explicitly rather than “produced,” since it is unclear and probably unlikely that any states produced such results intentionally in response to explicit mean improvement-disparity reduction targets, although Maryland set mean and disparity targets for a variety of health outcomes and determinants (20). Nor do we know the most cost-effective way of achieving the improvement some states achieved, either in any one of the measures or both together. Although we are beginning to collect evidence on effective programs and policies, such evidence often shows relationships for mean improvement rather than disparity reduction and overall effectiveness instead of cost effectiveness (21). Examining the states that performed well and states that performed poorly on both dimensions may reveal clues about the most effective policy packages to use for large improvement.

If a public or a private policy maker were interested in trying to determine what would produce optimal results, some standard of what “optimal” means would need to be defined. For this purpose, we believe it would be useful to have some summary metric of mean and disparity gap improvement such as the achievement index suggested by Wagstaff (22). As Wagstaff indicated, such a metric would have to reflect a value choice of the relative importance of mean improvement in mortality versus disparity reduction. Such a metric could be more complicated than the one he proposed, since several disparity domains also need to be considered. Since different states or communities would probably value each component (or each disparity domain) differently, a useful tool would be one that allows different weights to be used for each component so that achievement progress could be assessed component by component, perhaps beginning with a default standard that weighted components equally.

Despite these challenges, we believe that our results are useful now in beginning to set benchmarks for what is possible and to identify programs and policies that are most closely related to improved performance. We encourage public and private entities to 1) review what many states achieved in both general improvement and disparity reduction and 2) set policy and investment priorities in accordance with their own values and perspectives (23).
